# Gross motor function in children with Congenital Zika Syndrome from Rio de Janeiro, Brazil

**DOI:** 10.1007/s00431-021-04270-1

**Published:** 2021-10-01

**Authors:** Carla Trevisan M. Ribeiro, Tatiana Hamanaka, Sheila Pone, Mitsue Senra Aibe, Saint Clair Gomes, Karin Nielsen-Saines, Elizabeth B. Brickley, Maria Elisabeth Moreira, Marcos Pone

**Affiliations:** 1 National Institute of Women, Children and Adolescents Health Fernandes Figueira, Oswaldo Cruz Foundation (IFF-Fiocruz), Avenida Rui Barbosa, 716, Flamengo, Rio de Janeiro, 22250-020 Brazil; 2 David Geffen School of Medicine, University of California, Los Angeles, USA; 3 School of Hygiene & Tropical Medicine, London, UK

**Keywords:** Zika, Zika virus infection, Neurodevelopmental disorders

## Abstract

Congenital Zika Syndrome (CZS) is characterized by many impairments especially in the central nervous system, potentially compromising neurodevelopment and causing significant morbidity in affected children. The aim was to assess gross motor function in children with CZS. This was a cross-sectional investigation nested within a prospective cohort study of children with CZS based in a Brazilian referral hospital in Rio de Janeiro. Between March/2017 and February/2018, we performed gross motor function assessments using the Gross Motor Function Classification (GMFCS) and the Gross Motor Function Measure (GMFM), estimating the mean and standard deviation of GMFM scores among GMFCS groups. The study sample included 72 children, with a median age of 13 months (7–25). Of these, 63 (87.5%) had severe motor impairment, 3 (4%) had moderate impairment, and 6 (8%) had mild impairment. The mean GMFM score for each group was respectively 11.6, 26.1, and 81.6, with statistically significant differences (p-value < 0.001). Severely affected children only achieved head control in the sitting posture when supported. Children with milder forms were able to develop walking skills.

*Conclusion:* Most children with CZS have major motor disabilities and a poor prognosis. Better understanding of limitations and functionality in children with CZS can serve as a prognostic guide in their management.

**Table:** 

**What is Known:** *• Severe motor impairment was present in 63 (87.5%) children with CZS.* *• The degree of neurological impairment was inversely associated with motor performance.*	
**What is New:** *• Microcephaly was more frequent among children with severe gross motor function impairment.* *• Children with CZS have major motor disabilities and a poor prognosis.*

**What is Known:**

*• Severe motor impairment was present in 63 (87.5%) children with CZS.*

*• The degree of neurological impairment was inversely associated with motor performance.*

**What is New:**

*• Microcephaly was more frequent among children with severe gross motor function impairment.*

*• Children with CZS have major motor disabilities and a poor prognosis.*

Fetal infection by Zika virus (ZIKV) may disrupt brain embryogenesis and result in microcephaly and other neurological abnormalities, including subcortical parenchymal calcifications, ventriculomegaly, and cortical migration abnormalities [1, 2]. Congenital Zika Syndrome (CZS) is characterized by the presence of these central nervous system (CNS) abnormalities as well as osteoarticular deformities and hearing and/or visual impairments, which may compromise neurodevelopment and cause significant morbidity in affected children [1–5]. These sensory and motor alterations are similar to those present in children with cerebral palsy (CP) who have musculoskeletal and neural restrictions that impede the acquisition of functional abilities [6–8]. Motor changes in CZS are often associated with cognitive, communication, and behavioral disorders that prevent children from reaching developmental milestones [6, 7]. The Gross Motor Function Measure (GMFM) was designed and validated to assess changes in gross motor function over time in children with CP [9, 10] and it can be an efficient tool for assessing neurodevelopment of children with CZS. The Gross Motor Function Classification System (GMFCS) is used to define a child’s ability and functional limitations, serving as a guide to inform motor prognosis [11]. The purpose of this study was to assess gross motor function in children with CZS, a recently described clinical condition with scarce data available on the neurodevelopmental trajectories of affected populations.

## Methods

The study was conducted at the National Institute of Women, Children and Adolescents Health Fernandes Figueira, Oswaldo Cruz Foundation (IFF-Fiocruz), a major referral center for congenital infections in Rio de Janeiro, Brazil. Infants with presumed CZS were enrolled in a longitudinal cohort during the ZIKV outbreak in Brazil in 2015–2016. The study population was comprised of children with CZS who were evaluated in this study once between the ages of 7 and 25 months from March 2017 to February 2018. This cross-sectional study nested within a cohort was approved by the IFF-Fiocruz institutional review board (Plataforma Brasil, CAAE: 52,675,616.0.0000.5269). Parents or guardians provided written informed consent for their children’s participation.

The study sample included children, older than 6 months, born during or immediately after the ZIKV outbreak in Rio de Janeiro, Brazil. Children with presumed/probable CZS were defined as having characteristic clinical features, neurological manifestations, neuroimaging abnormalities, and/or ocular alterations typical of congenital ZIKV infection, assessed by neurofunctional physical therapy. Children with confirmed CZS had all the features of children with presumed/probable CZS plus laboratory confirmation of maternal or fetal infection. The characteristic clinical phenotype of CZS included: microcephaly, overlapping cranial sutures, partially collapsed skull, prominent occipital bone, redundant scalp skin, and/or severe neurological impairment, with or without accompanying arthrogryposis. Neurological manifestations included: irritability, hypertonia and spasticity, hypotonia, hyperreflexia, and/or seizures. The alterations considered in the neuroimaging exams included: intracranial calcifications, ventriculomegaly and extra-axial fluid, abnormal gyral pattern (e.g., polymicrogyria), decreased cerebral parenchymal volume, cortical atrophy and malformations, cerebellar or cerebellar vermis hypoplasia, delayed myelination, and hypoplasia of the corpus callosum. The typical ocular alterations included: hypoplasia or pallor of the optic nerve, increased cup-to-disc ratio, chorioretinal atrophy or scar, pigment mottling, hemorrhagic retinopathy and abnormal retinal vasculature.

Children with arthrogryposis or other osteoarticular congenital malformations were excluded from the analysis to reduce confounding factors in motor assessment. Other congenital infections within the TORCH group, such as toxoplasmosis, rubella, cytomegalovirus infection, and herpes simplex virus infection were excluded by negative serology. Microcephaly was defined as head-circumference at birth below -2 standard deviations for gestational age and gender (z-score -2). Similarly, severe microcephaly was defined as a head-circumference at birth below z-score -3. We used the INTERGROWTH-21st Project online software to calculate Z-scores for birth measurements based on gestational age and sex (http://intergrowth21.ndog.ox.ac.uk) [12–14].

Each child was evaluated once by a GMFM-trained physical therapist (ICC > 0.98 for trained) and was classified according to the GMFCS-88. We assessed 88 items in the following dimensions: (A) Lying and rolling; (B) Sitting; (C) Crawling and kneeling; (D) Standing; and (E) Walking, running, and jumping. Children were also classified in 5 progressive levels of independence and functionality by GMFCS total score. Level I indicates the ability to walk without any restrictions. Level II indicates some limitations in gait. At level III, children need some assistance to walk. At level IV, children need assistive technology equipment to move. Level V is reserved for children with severe movement limitations even with the aid of modern technology and total dependence for performance of routine tasks. For analysis purposes, we classified children at levels I and II as having mild impairment, at level III as having moderate impairment, and at levels IV and V as severe impairment; and the GMFM were described quantitatively according to the standardization of the scale with percentage of each dimension and total percentage. Data were analyzed as the mean and standard deviation of these percentages according to the groups of functional impairment [15–17]. Additional information on age, sex, head circumference at birth and prenatal ZIKV infection confirmation (i.e., maternal infection during pregnancy or congenital infection confirmed at birth) was extracted from medical records.

Data was stored in an EPIINFO 7 database. The data analysis was descriptive with measures of central tendency and position and measures of dispersion. GMFM results were analyzed by the Gross Motor Ability Estimator software (GMAE). Analysis of gross motor function occurred through the mean and standard deviation by disease severity groups. Kruskal–Wallis tests were used to test for differences between groups for continuous variables and chi-square tests were used for categorical variables. The Spearman correlation index was used to verify the association between the GMFCS functional classification categories and the GMFM-88 total scores (percentage). Statistical Package for Social Sciences (SPSS) version 20.0 was used, and the significance level of α = 0.05 was considered.

## Results

Seventy-two children with CZS were evaluated, of whom 39 (54%) were male, with ages ranging from 7 to 24 months (median age 13 months). Prenatal ZIKV infection was confirmed via real-time reverse-transcriptase–polymerase-chain reaction (RT-PCR) assays in 31 (43%) cases for either the mother, neonate, or both.

Most children, 63 (88%) cases, had severe gross motor impairment, 3 (4%) had moderate impairment, and 6 (8%) had mild impairment. There was no significant difference between groups regarding age and sex (Table 1). Microcephaly was more frequent in the severe gross motor function impairment group, occurring in 51 (83%) cases, with a statistically significant difference between groups (p-value = 0.001, Chi-squared test). In fact, 35 (56%) children from this group presented with severe microcephaly. Nevertheless, microcephaly was also observed in 2 children with mild gross motor function impairment. Most children underwent physical therapy and started treatment by a mean age of 4.7 months (range: 1 to 16 months; SD: 3.0).

**Table 1:** Description of study population according to GMFCS classification as mild, moderate, and severe impairment groups

		**GMFCS**			**Total**
		Mild	Moderate	Severe	
**Number of participants**		n = 6	n = 3	n = 63	N = 72
**Mean (SD) age in months**		14.5 (1.4)	11.7 (0.6)	14.1 (4.7)	13.9 (4.4)
**Sex**	Female	3 (50.0%)	1 (33.3%)	29 (46.0%)	33 (45.8%)
	Male	3 (50.0%)	2 (66.7%)	34 (54.0%)	39 (54.2%)
**Microcephaly**	Yes	2 (33.3%)	0	51 (81.0%)	53 (73.6%)
	No	4 (66.7%)	3 (100%)	12 (19.0%)	19 (26.4%)
**Microcephaly severity**	Moderate	1 (50.0%)	0	16 (31.4%)	17 (32.1%)
	Severe	1 (50.0%)	0	35 (68.6%)	36 (67.9%)
**ZIKV detection** **(RT-PCR)**	Yes	4 (66.7%)	2 (66.7%)	25 (39.7%)	31 (43%)
	No	2 (33.3%)	1 (33.3%)	38 (60.3%)	41 (57%)
**Physical therapy**	Yes	4 (66.7%)	3 (100%)	56 (88.9%)	65 (87.5%)
	No	2 (33.3%)	0	7 (11.1%)	9 (12.5%)
**GMFCS levels**		I = 0 II = 6	II = 3	IV = 42 V = 21	–
**Mean (SD) GMFM**		81.6 (5.9)	26.1 (7.0)	11.6 (3.6)	17.6 (20.2)

GMFCS, gross motor function classification system; RT-PCR, reverse transcription-polymerase chain reaction; ZIKV, Zika virus

The mean GMFM score was 81.6 (ranging from 75 to 90) in the mild impairment group, 26.1 (19 to 33) in the moderate impairment group and 11.6 (4 to 22) in the severe impairment group. There was a statistically significant difference between groups (p-value < 0.001, ANOVA test). The mean total GMFM score was 17.6 and the median (range) score was 11.5 (3.7–90.3). Only 9 (12.5%) children did not undergo physical therapy. Spearman correlation index revealed a negative and statistically significant association between GMFCS groups and GMFM score (−0.6; p-value < 0.001).

Regarding GMFM results, the severely impaired group, which corresponded to 88% of the children, was functionally limited and therefore only scored in the first two dimensions for (A) lying and rolling (mean score = 38.8), and (B) sitting (mean score = 16.8). In comparison, children in the moderately impaired group had higher scores in the second dimension (sitting), with many being able to remain seated without upper limb support, and some even had some abilities on dimension C (crawling and kneeling), with a mean score of 4.0 in this dimension. On the other hand, children in the mildly impaired group had fewer limitations in their gross motor skills, and a subset were able to reach some abilities of dimension E (walking, running and jumping), with a mean score of 36.4 (Table 2).

**Table 2:** Mean (SD) GMFM scores in each dimension according to gross motor function impairment groups

	**Mild** n = 6	**Moderate** n = 3	**Severe** n = 63
Dimension A (lying and rolling)	100 (0)	84.3 (16.7)	38.3 (14.9)
Dimension B (sitting)	98.6 (2.2)	42.2 (12.5)	16.8 (6.2)
Dimension C (crawling and kneeling)	90.0 (11.3)	4.0 (6.9)	0 (0)
Dimension D (standing)	83.3 (9.3)	0 (0)	0 (0)
Dimension E (walking, running, and jumping)	36.4 (15.7)	0 (0)	0 (0)

Means and standard deviation in parentheses

## Discussion

The study results demonstrate an association between the degree of neurological impairment, as indicated by GMFCS level, and gross motor performance in children with CZS, as previously described in the CP literature [8, 12, 18–20]. As described in CP studies [15, 21], children with severe impairment (GMFCS levels IV and V) also had severe impairment of gross motor function, with a low mean GMFM score. Severely impaired children in our study were unable to achieve more advanced postures (such as sitting, crawling and walking), with some only reaching head control in the sitting posture when supported by the therapist. Also of note, despite neuromotor impairments, some children with milder forms of CZS, especially regarding muscle tone, were able to reach gait and other more elaborate functions in orthostatic posture, such as leaping and kicking a ball.

Using the GMFCS tool, 88% of children were classified in levels IV or V, indicating a more severe motor impairment and the need for assistance for performance of daily tasks. Such impairment was similar to that described in other case series of CZS [22–24]. In a study with 77 children with CZS, [22] observed that 97.4% (n = 75) had severe functional impairment (GMFCS IV and V) and only 2.6% (n = 2) had mild functional impairment (GMFCS I); however, the inclusion of children with arthrogryposis may have increased the frequency of serious abnormalities. Carvalho et al. [23] evaluated 69 children with CZS, of whom 4 had arthrogryposis, and observed that only 7% (n = 5) were classified as GMFCS I to III and 93% (n = 64) were classified as GMFCS IV or V. A recent study [24] with 110 children born with CZS had similar findings, where 90.2% of children had GMFCS V, 4.9% had GMFCS IV, 2% GMFCS III and 2.9% GMFCS I. The present study did not include children with arthrogryposis in order to avoid interference in GMFM scoring. The literature has already described the association between congenital orthopedic abnormalities and worse neurological outcome [25–27].

Children with CZS and muscle tone, sensory (visual and/or auditory) or orthopedic (hip dislocation and/or arthrogryposis) abnormalities have greater limitations in performance of daily tasks and are restricted in their ability to participate in age-appropriate activities. This can be further compounded by limited resources restricting access to medication, physical therapy or assistive technology [8]. Ferreira et al. [7] described a variety of structural and functional deficiencies in children with CZS, such as dysphagia, cognitive and language disorders, altered muscle tone, decreased control of voluntary movements and decreased joint mobility. These deficiencies deeply impacted performance of daily activities, as fine motor development was not age-appropriate in more than 90% of the children, who were also unable to walk. Another study by [19] analyzed 24 children with CZS and identified hyperreflexia in 100% of the sample, increased muscle tone in 95% and clonus in 77%. No child was able to stand or walk even with support, which denotes a significant motor delay. The present study results have demonstrated similar findings.

In the current study, the mean GMFM score was 11.5 (range: 3.7–90.3). Furthermore, there was an inverse association between GMFCS groups and GMFM scores, indicating the higher the level of GMFCS (greater functional impairment), the lower the GMFM score. Melo et al. [18] analyzed a group of children with CZS between 5 and 29 months of age and showed a mean GMFM score of 6.5 (range: 2–82.9), which is lower than that observed in this study.

GMFM and GMFCS are promising tools for improving the understanding of limitations and prognosis of children with CZS. They serve as a guide for tailoring care and informing health care and educational policies and priorities [10, 12]. The poor motor prognosis in our study participants raises concerns about prospects for the group’s full participation in the daily activities of society without substantial long-term support.

We used GMFM-88 instead of the reduced version of the scale, GMFM-66, since the full version is more sensitive for detection of alterations in dimensions A and B [28]. Younger and/or severely affected children are mostly able to reach developmental milestones in the first two dimensions; the research study sample was evaluated at a young age (mean of 13.9 months) and was mostly considered severely impaired. Therefore, GMFM-88 appears to be an appropriate tool to evaluate gross motor function in children with CZS.

As seen in Table 2, the group with severe impairment according to GMGCS levels, scored only in the first two dimensions (A) lying and rolling and (B) sitting, which demonstrates that they had a worse prognosis, with difficulties in performing simple tasks independently. Children in the group with moderate impairment managed to remain seated without support from the upper limb and even had some skills in dimension C (crawling and kneeling). These children have the ability to perform postural shifts with greater independence, but they may present postural compensations, like to sit with lumbar hyperkiohosis [23, 29]. Children in the group with mild impairment managed to score in all dimensions, and some of them were already walking. These results are expected, especially for those in the severe group, and have been observed in other studies of children with CZS [6, 8, 22].

The presence of microcephaly at birth is another important factor for the impact of ZIKV congenital infection on neurological development [3, 6, 19, 30, 31]. Gordon-Lipkin et al. [32] have demonstrated that congenital microcephaly is associated with gross motor development abnormalities in 65% of cases [31]. Microcephaly in CZS is caused by a disruptive process [7, 13]. Severe microcephaly is caused by brain growth arrest, mainly affecting the frontal, temporal and parietal lobes [7, 32]. There is a close relationship between neuromotor development delay and the degree of microcephaly, as the impaired head growth is indicative of the extent of brain damage [2, 8, 33, 34]. The study results corroborate these findings, since severe microcephaly was more frequent in the group of children classified as severely impaired by GMFCS.

It is important that longitudinal cohort studies evaluating sensorineural development use standardized scales. A recent study in the northeastern region of Brazil was the first to analyze motor development of children with CZS over time; this study reported advances in motor development in the first 18 months of age [22]. However, at 24 months of age, most children had reached 90% of their final motor abilities [22].

Although the present study was cross-sectional, prospective studies of children with CZS are essential for the understanding of the natural history of congenital ZIKV infection, motor development of affected children, and their functional impairments. Although exclusion of children with arthrogryposis can be considered a study limitation in the present study, their inclusion would have negatively impacted gross motor development results in CZS. Another limitation the asymmetrical distribution of participants between groups, which may have affected our statistical analysis.

## Conclusion

Low mean GMFM scores in this population, where most children were classified as GMFCS IV and V, demonstrate the severe functional impairment and poor prognosis of children born with CZS. Our results highlight the pressing need for attention to the neurodevelopment of this population with the implementation of early stimulation programs, public health policies and inclusive education, in order to reduce the burden of disease and allow for better motor and sensory development in affected children.
